# Maternal Psychological Distress and Placental Circulation in Pregnancies after a Previous Offspring with Congenital Malformation

**DOI:** 10.1371/journal.pone.0086597

**Published:** 2014-01-27

**Authors:** Anne Helbig, Anne Kaasen, Ulrik Fredrik Malt, Guttorm Haugen

**Affiliations:** 1 Norwegian Resource Centre for Women's Health, Oslo University Hospital, Oslo, Norway; 2 Department of Obstetrics, Oslo University Hospital, Oslo, Norway; 3 Department of Health, Nutrition and Management, Oslo and Akershus University College of Applied Sciences, Oslo, Norway; 4 Department of Neuropsychiatry and Psychosomatic Medicine, Oslo University Hospital, Oslo, Norway; 5 Institute of Clinical Medicine, University of Oslo, Norway; Scuola Superiore Sant'Anna, Italy

## Abstract

**Introduction:**

Antenatal maternal psychological distress may be associated with reduced placental circulation, which could lead to lower birthweight. Studies investigating this in humans show mixed results, which may be partially due to type, strength and timing of distress. In addition, the arterial vascular resistance measures often used as outcome measures do not detect smaller changes in placental volume blood flow. We aimed to investigate the effect of a specific stressor, with increased levels of stress early in pregnancy, on the fetoplacental volume blood flow in third trimester.

**Methods:**

This was a prospective observational study of 74 pregnant women with a congenital malformation in a previous fetus or child. Psychological distress was assessed twice, around 16 and 30 weeks' gestation. Psychometric measures were the General Health Questionnaire-28 (subscales anxiety and depression), Edinburgh Postnatal Depression Scale, and Impact of Event Scale-22 (subscales intrusion, avoidance, and arousal). Placental circulation was examined at 30 weeks, using Doppler ultrasonography, primarily as fetoplacental volume blood flow in the umbilical vein, normalized for abdominal circumference; secondarily as vascular resistance measures, obtained from the umbilical and the uterine arteries.

**Results:**

Maternal distress in second but not third trimester was associated with increased normalized fetoplacental blood flow (P-values 0.006 and 0.013 for score > mean for depression and intrusion, respectively). Post-hoc explorations suggested that a reduced birthweight/placental weight ratio may mediate this association. Psychological distress did not affect vascular resistance measures in the umbilical and uterine arteries, regardless of adjustment for confounders.

**Conclusions:**

In pregnant women with a previous fetus or child with a congenital malformation, higher distress levels in second trimester were associated with third trimester fetoplacental blood flow that was higher than expected for the size of the fetus. The results do not support placental blood flow reduction as a pathway between maternal distress and reduced birthweight.

## Introduction

The possible unfavorable effects of antenatal maternal psychological distress on the developing fetus are an area of growing scientific interest. In humans, this type of research is, by necessity, mainly observational. This leads to considerable methodological challenges through variations in type, timing and duration of exposure, as well as in biological, behavioral and psychosocial characteristics of the population and the individual. Various types of maternal distress including psychosocial stress, anxiety, and depression, have been suspected of negatively affecting birthweight [1]. However, findings from human studies are not consistent. Recent reviews and well-controlled prospective population-based studies have concluded that some types of maternal distress seem to be associated with a small reduction in average birthweight (i.e. 20–50 g) [2]–[5]. Although hardly of clinical importance when seen in isolation, this physiological effect could still be relevant in the complex interaction between the maternal physical and psychosocial environment, the placenta, and the fetus.

The mechanism for an association between maternal distress and birthweight is still unclear. As the placenta plays a major role in the control of fetal growth and development, several studies have investigated the association between maternal stress and the placental circulation. Although several studies found increased placental vascular resistance [6]–[8], the majority of reports do not support these findings [9]–[13]. As with other studies on maternal distress in pregnancy, these discrepancies could partially be due to considerable methodological differences such as the population examined, the type and severity of psychological distress, the gestational timing of distress and circulatory assessment, and the circulatory measures chosen. Placental vascular resistance is assessed non-invasively in the uterine (UtA) and umbilical (UA) arteries using Doppler ultrasound-derived blood flow velocity measurements. Resistance indices such as the pulsatility index (PI) consist of ratios calculated from blood flow velocities (i.e. cm/s) in different parts of the heart cycle (e.g. systole and diastole). However, while easy to obtain, UtA and UA resistance indices are semi-quantitative and their ability to detect smaller changes in the placental circulation is limited [14]. The umbilical vein (UV), as the single vessel carrying blood from the placenta to the fetus, is uniquely suited for volume flow measurements (i.e. ml/min), thereby providing more direct physiological information. Our previous report was the first to examine fetoplacental volume blood flow in relation to antenatal distress in women with uncomplicated obstetric histories [13]. We found a possible reduction of blood flow in third trimester in emotional distress regarding the health of the fetus. However, this reduction was not present for symptoms of depression or anxiety. Since this was an observational non-randomized study with a single positive result, we discussed the need for corroboration. Assuming there might be a dose-response effect, we suggested that a study group with high levels of emotional distress about the health of the fetus would be of particular interest.

In the current study, we recruited pregnant women with a congenital malformation in a previous fetus or child. These women were at high risk of psychological distress about the health of the fetus [15], [16]. According to a recent review, depression is the psychological state most consistently associated with reduced birthweight [17]. We used the same design as before to investigate the association between antenatal distress in third trimester and the placental circulation. In addition, we examined the role of maternal distress in the first half of pregnancy on third trimester placental circulation. Due to the rapid development of the materno-placental interface and the fetal cardiovascular system, the early phase of pregnancy may represent a particularly vulnerable period to the influence of significant maternal stress [18], [19]. Early changes in circulatory structure or function could persist; while not leading to immediate measurable effects, their influence might become detectable later in pregnancy [3].

We hypothesized that distress would have a stronger effect on the placental circulation in third trimester when occurring earlier in pregnancy. Based on previous results, we expected that distress in second and possibly third trimester would primarily affect UV volume blood flow, but not – or to a lesser extent – UA and UtA vascular resistance indices.

## Materials and Methods

### Ethics statement

The study was approved by the Regional Committee for Medical Research Ethics, Southern Norway, Oslo, Norway (S-05281). Written informed consent was obtained from all participants, and the study was conducted in accordance with the Declaration of Helsinki.

### Methods

The present study is part of a larger prospective study investigating psychological distress in three groups of pregnant women, with either a current diagnosis of fetal malformation, malformation in previous offspring, or no history of congenital malformation. The present study describes only the group with previous malformations in a fetus or child. Design, choice of psychometric tools, and sample sizes of the different groups were determined with regard to the larger study [20].

### Participants

The study group consisted of 80 pregnant women with a history of a structural malformation in a previous fetus or child, considered to be detectable antenatally by an experienced ultrasonographer. Between May 2007 and October 2009, the participants were referred to our tertiary centre for fetal medicine because of their history. They were invited in second trimester after ultrasound examination had shown normal results, regardless of whether further ultrasound examinations later in pregnancy were deemed necessary. Previous malformations ranged from lethal to moderate, detected either before or after birth, and included aneuploidies if accompanied by structural malformations. Previous pregnancy outcomes ranged from termination of pregnancy in more than half of the study group, to living children without persisting disabilities (n = 10). Inclusion was done consecutively, but with limitations imposed by workload (i.e. convenience sampling). Exclusion criteria were multiple pregnancy, insufficient fluency in Norwegian, overt psychiatric disorders (e.g. severe bipolar disorder, psychosis, drug abuse), and age below 18 years.

The first assessment (T1) was performed as soon as possible after consent was given, i.e. mostly around 16 weeks. This included psychometric questionnaires, ultrasound examination with fetal biometry, and measurement of maternal weight and blood pressure. The assessment was repeated at 30 weeks gestation (T2), including Doppler ultrasound to assess the placental circulation. Sociodemographic, medical, and obstetric data including postpartum follow-up, neonatal and placental weight were systematically collected by interviews, self-report questionnaires, from medical records, and from the report form for the National Birth Registry. The hospital where delivery took place was determined by geographical and patient preference considerations, with 40 participants delivering at our hospital. Norwegian reference charts were used to calculate the standard deviation (SD) scores for gestational age and gender-adjusted neonatal and placental weight [21], [22]. Maternal body mass index (BMI; kg/m^2^) was calculated using self-reported height.

### Psychometric measures

Psychological distress was assessed by three self-report questionnaires: the 28-item version of the General Health Questionnaire (GHQ) [23], the Edinburgh Postnatal Depression Scale (EPDS) [24] and the 22-item Impact of Event Scale (IES) [25], [26]. These scales measure core dimensions of psychological distress, i.e. emotional, cognitive, physiological, and behavioral symptoms.

The GHQ-28 has been widely used to estimate the prevalence of mental disorders in a given population, but also as a measure of psychological distress and subjective well-being in clinical and non-clinical populations. It has also been used in relation to pregnancy and offspring congenital malformations [13], [16], [20], [27]. All items have four possible responses and emphasize the last two weeks. Likert scoring (item scores 0-1-2-3, sum score range 0–84) is mostly used as an ordinal scale to measure level of distress. The mean score in a given population is suggested to be indicative of the best threshold when estimating prevalence of probable clinically significant levels of distress [28], although other authors suggest other parameters [29]. When using the case score method (0-0-1-1 scoring per item, total range 0–28) to identify clinically significant distress, a score ≥6 is often used.

Factor analyses of the GHQ-28 have identified four subscales, with seven items each (Likert scoring range of 0–21). The “somatic symptoms” subscale covers general health issues, while “social dysfunction” focuses on general well-being and quality of life. The anxiety subscale addresses symptoms such as sleep problems, nervousness, and panic attacks. The depression subscale deals with rather severe symptoms such as hopelessness and suicidal ideation. The subscales are mostly used as ordinal scales to measure severity of symptom dimensions, not as case-finders for clinical depression or anxiety. We considered the anxiety and depression subscales to be the most relevant for our specific research question.

The EPDS is a 10-item scale originally designed as a screening tool for postpartum depression, but has been validated for use during pregnancy [30]. It refers to the previous week, with a scoring range of 0–3 per item (range 0–30). A cut-off value of 10 has been found to have good psychometric properties for identifying clinically significant depressive symptoms in a Norwegian validation study [31]. EPDS describes less severe depressive symptoms than the GHQ-28 depression subscale. EPDS has been used to describe parental psychological response to fetal malformation, and to subsequent termination of pregnancy [15], [20].

While neither GHQ-28 nor EPDS address psychological reactions to a specific stressor, the IES measures symptoms of distress in relation to a defined stressful or traumatic event. It has good psychometric properties in both clinical and non-clinical samples [32] and has been used in several studies on diagnosis of fetal congenital malformations and related fetal loss [15], [16], [20], [27]. In the present study the questionnaire referred to “the condition of the child” as a possible cause for distress. The IES-22 consists of 22 items (scoring range 0–5 per item), referring to the previous week, and has three subscales. Intrusion (seven items, range 0–35) deals with symptoms such as intrusive and unbidden thoughts, emotions, dreams, and memories. Avoidance (seven items, range 0–35) addresses emotional numbness, denial, and avoiding stimuli or thoughts related to the health of the fetus. Arousal (eight items, range 0–40) focuses on psycho-physiological symptoms such as hypervigilance, irritability, and heightened startle response. In clinical samples, subscale scores below 9 usually indicate minor responses, 9–19 moderate responses, and above 19 clinically important responses.

Our sample was relatively small, and adhering to clinically defined cut-off levels would have led to a significant loss of power. In addition, physiological changes may occur within ranges considered normal. As in our previous study, we chose to use a cut-off level at the mean for all psychometric measures in addition to ordinal scores [13].

### Ultrasonography

Ultrasound examinations were done immediately after completion of the questionnaires, by an experienced operator (AH or GH) using an Acuson Sequoia 512 ultrasound machine (Mountain View, CA, USA) with a transabdominal 2.5–6 MHz curvilinear probe. Abdominal circumference, UV diameter, and blood flow velocity waveforms of the intra-abdominal UV, one UA, and both UtAs were obtained with participants in a semi-recumbent position. Flow velocity waveforms were acquired using pulsed-wave Doppler with color Doppler guidance. The high pass filter was set at 50 Hz. The sample volume was adjusted to encompass the diameter of the vessel. The lowest possible insonation angle was used, always below 25 degrees, with angle correction. Images were stored digitally and analyzed off-line by the ultrasonographer, blinded to distress scores. Acoustic output and exposure time was kept as low as reasonably achievable.

UV flow velocity waveforms were obtained from the intra-abdominal straight part in the fetal liver during fetal quiescence. Steady state waveforms of 2–4 seconds duration were traced manually, and the time-averaged maximum velocity (V_TAMX_, cm/s) recorded. UV inner diameter was measured at the same site, perpendicular to the vessel wall, in memory buffer frames with the best visualization of the vessel walls. The mean was calculated from at least five measurements [33]. Fetal abdominal circumference (AC, cm) was measured in a standardized transverse view and recorded as the mean of three measurements. Q_UV_ (ml/min) was calculated as 0.5 * V_TAMX_ * π * (UV diameter/2)^2^ * 60 and divided by fetal AC to normalize for fetal size (Q_UVAC_, ml/min/cm) [34]. Normalizing for AC instead of estimated fetal weight was chosen in order to minimize measurement errors by using a single rather than a composite measure [14], [34], [35].

The UA was assessed in a free loop of the umbilical cord during fetal quiescence. For the UtA, the sample gate was placed within 1 cm ventrally of the cross-over with the external iliac artery. Three consecutive blood flow velocity waveforms in steady state were manually traced and their mean PI calculated. For the UtA, the average of both sides was used. Fetal and maternal heart rates were obtained as an average of three waveforms from the UA and the right UtA, respectively.

### Statistical analysis

The primary outcome variable was Q_UVAC_, secondary outcome variables were UA PI and UtA PI. Explanatory variables were the different psychometric scores, ordinal as well as dichotomized at the mean. Covariates selected *a priori* for regression models were gestational age, maternal age, parity (para 0/≥1), assisted fertilization (yes/no), smoking (yes/no), BMI, fetal gender, fetal and maternal heart rates, and ultrasonographer. In post-hoc analyses for Q_UVAC_, UA PI, birth- and placental weight SD-scores, as well as birthweight/placental weight (Bw/Pw) ratio were explored as covariates. For descriptive statistics and comparisons between groups, parametric or non-parametric analyses were used as appropriate. Q_UVAC_ and UtA PI were not normally distributed and ln-transformed if this allowed the use of parametric methods or to optimize residual plots in linear regression models. Bivariate associations between variables were explored by T-tests or Mann-Whitney *U* test, and scatter plots with Pearson's or Spearman's correlation coefficients, as appropriate. For linear regression analyses, subscale scores and other covariates were first examined in univariate analyses. Covariates with *P<0*.20 were entered stepwise into multiple regression models, and variables with an adjusted *P<0*.10 were kept in the final models. For analyses with Q_UVAC_ as outcome, our selection criterion of *P<0*.20 in univariate analyses applied to all psychometric subscales except avoidance. In addition, some subscales were highly inter-correlated. We therefore selected two key measures for further analyses: Intrusion had been previously defined as being of interest given the results of our previous study. Depression was least correlated with the other relevant subscales; it also has been identified as most consistently associated with reduced birth weight [17]. Model assumptions regarding normality, linearity, outliers, homoscedasticity, and independence of residuals were checked. Sample size was determined a priori by the larger study which this subset of women participated in [20]. The current sample was sufficient to detect a difference of approximately 0.75 SD between two similarly sized groups with a power of 90% and a 5% significance level. The increase in UtA resistance index found by Teixeira *et al.* in a low-risk population corresponded to 1 SD [7]. Due to the inability to obtain measurements in all cases (see Results), the corresponding detectable effect size for Q_UVAC_ was 0.82 SD. Data analysis was performed using SPSS version 18 (Statistical Package for the Social Sciences, Chicago, IL, USA).

## Results

Of the 80 women originally recruited, one withdrew before T2. Five were excluded before statistical analysis due to maternal pre-existing conditions or medications known to affect fetal growth or circulation. There were no cases of gestational diabetes.

Population characteristics and obstetric outcome of the remaining 74 participants are described in Table 1. Placental weight was available in 70 cases.

**Table 1:** Demographic characteristics and obstetric outcome data.

Characteristic	n = 74
Maternal age (years)	32.1±4.5
Parity ≥1	58
Maternal BMI at 30 weeks	27.7±4.3
Smoking (yes)	5
Assisted fertilization (yes)	4
Gestational age at first assessment (weeks)	15.7 (14.1–18.2)
Gestational age at second assessment (weeks)	30.0±0.6
Gestational age at delivery (weeks)	39.8±1.5
Preterm birth (<37 weeks)	2^a^
Pregnancy induced hypertension/preeclampsia	5^b^
Birthweight (g)	3524±476
Placental weight (g)^c^	706±148
Birthweight/placental weight ratio^c^	5.12±0.86
Fetal gender (female)	39

Data are given as mean ± SD, median (interquartile range) or *n*. BMI, body mass index, kg/m^2^.

a One fetal death.

b One pregnancy-induced hypertension at 30 weeks, all others late onset.

c n = 70.

Psychometric scores are presented in Table 2. At T1, between 20 and 60% of participants scored above established cut-off levels indicating significant distress (GHQ case score ≥6), possible depression (EPDS ≥10), or severe intrusive symptoms (IES intrusion ≥20) (see Table S1, Psychological distress scores above clinical cut-off levels at 16 and 30 weeks of gestational age). At T2, all scores were lower than at T1, but still considerably higher than in women without previous malformations [13].

**Table 2:** Psychological ordinal distress scores at 16 and 30 weeks of gestational age.

	T1 (16 weeks), n = 73^a^		T2 (30 weeks), n = 74	
	Mean (SD)	Median (Range)	Mean (SD)	Median (Range)
GHQ–28				
Sum Likert score	24.3 (9.1)	23 (8–53)	21.4 (9.5)	21 (8–52)
Sum case score	7.0 (5.0)	7.0 (0–19)	5.2 (5.0)	3.0 (0–21)
Anxiety	7.3 (3.7)	7 (1–14)	6.2 (3.3)	6 (1–14)
Depression	1.1 (2.2)	0 (0–13)	0.6 (1.6)	0 (0–8)
Somatic symptoms	7.0 (3.8)	6 (1–17)	6.0 (3.6)	5 (1–18)
Social dysfunction	9.0 (2.7)	8 (0–14)	8.6 (2.5)	8 (3–15)
EPDS				
Sum score	5.8 (4.2)	6 (0–18)	4.6 (4.0)	3 (0–15)
IES				
Intrusion	16.2 (7.6)	17 (0–34)	11.2 (8.3)	10 (0–32)
Avoidance	7.8 (7.7)^b^	5 (0–35)^b^	4.6 (5.3)	3 (0–24)
Arousal	8.8 (6.8)^b^	8 (0–32)^b^	6.5 (6.4)	5 (0–24)

a Missing n = 1 (missing set of questionnaires).

b n = 72 (1 missing set of questionnaires; 1 page of questionnaire left blank).

EPDS, Edinburgh Postnatal Depression Scale; GHQ, General Health Questionnaire; IES, Impact of Event Scale.

### Umbilical vein volume blood flow

Measurement of Q_UV_ (median 133.7 ml/min, interquartile range 111.8–172.6) and Q_UVAC_ (median 5.04 ml/min/cm, interquartile range 4.23–6.27) at 30 weeks was successful in 66 participants.

For maternal distress assessed at T1, Q_UVAC_ was significantly correlated with ordinal EPDS, intrusion and arousal scores (see Table 3 and Figure S1, Association between IES intrusion score and normalized UV volume blood flow). Borderline significance was present for depression scores (*P* = 0.07). Increasing distress levels were associated with higher volume blood flow. Q_UVAC_ was significantly higher in participants scoring above the mean for depression, EPDS, intrusion, or arousal (see Table 3).

**Table 3:** Associations between second trimester psychological distress scores at 16 weeks and third trimester normalized umbilical vein volume blood flow (n = 65).

	Ordinal distress scores^a^		Dichotomous distress scores (cut-off level at the mean)^b^			
			score ≤ mean	score > mean		
	r_s_	P	Q_UVAC_,	Q_UVAC_,	n below	P
			median (IQR)	median (IQR)	vs. above	
GHQ Anxiety	0.18	0.15	4.67 (3.96–5.81)	5.44 (4.53–6.93)	32/33	0.10
GHQ Depression	0.23	0.070	4.86 (4.08–5.85)	5.87 (4.92–7.79)	49/16	0.008
EPDS	0.31	0.013	4.71 (4.05–5.69)	5.51 (4.54–6.93)	31/34	0.026
IES Intrusion	0.27	0.030	4.70 (2.90–5.73)	5.44 (4.67–6.99)	30/35	0.017
IES Avoidance^c^	0.14	0.29	4.92 (4.08–6.31)	5.21 (4.43–6.99)	37/27	0.26
IES Arousal^c^	0.31	0.012	4.85 (3.86–5.40)	5.58 (4.53–7.36)	31/33	0.011

a Correlations (Spearman's correlation coefficient) between ordinal psychological distress scores at 16 weeks of gestational age and umbilical vein volume blood flow (Q_UVAC_, normalized for fetal abdominal circumference, ml/min/cm) at 30 weeks.

b Mann-Whitney *U* test comparing Q_UVAC_ (median, interquartile range) at 30 weeks of gestational age for women scoring below *vs.* above the mean for psychological distress at 16 weeks.

c Missing n = 1 (page of questionnaire left blank).

EPDS, Edinburgh Postnatal Depression Scale; GHQ, General Health Questionnaire; IES, Impact of Event Scale; IQR, inter-quartile range; Q_UVAC_, umbilical vein volume blood flow normalized for fetal abdominal circumference (ml/min/cm); r_s_, Spearman's rank correlation coefficient.

As outlined in the methods section, we then selected intrusion and depression subscale scores for further examination in multiple regression models. Covariates were added as previously described, but none reached the required adjusted *P<0*.10 in any of the models. Consequently, in the regression analyses the only significant predictors of ln-Q_UVAC_ were ordinal intrusion scores, as well as intrusion and depression above the mean (see Table 4). The regression coefficient for the dichotomous variables corresponded to 0.61 and 0.77 SD of ln-Q_UVAC_, and variance explained was between five and ten percent.

**Table 4:** Linear regression exploring the role of birthweight/placental weight ratio on the association between distress measures and ln-transformed normalized umbilical vein blood flow.

	unadjusted^a^			adjusted for Bw/Pw ratio^a^		
	B^b^	95% CI	P	adj. B^b^	95% CI	P
T1 GHQ depression (ordinal)	0.024	−0.007, 0.054	0.12	0.015	−0.015, 0.045	0.33
T1 GHQ depression > mean	0.222	0.065, 0.380	0.006	0.160	−0.007, 0.326	0.060
T1 IES intrusion (ordinal)	0.010	0.001, 0.019	0.036	0.008	−0.002, 0.017	0.10
T1 IES intrusion > mean	0.176	0.038, 0.313	0.013	0.132	−0.010, 0.275	0.068

Results from linear regression analyses showing associations between ordinal and dichotomous distress measures at 16 weeks gestational age and ln-transformed umbilical vein blood flow normalized for fetal abdominal circumference (Ln-Q_UVAC_, ml/min/cm) at 30 weeks, with adjustment for birthweight/placental weight ratio.

a Distress measures n = 65, Bw/Pw ratio n = 61.

b B, regression coefficient; Bw/Pw, birthweight/placental weight.

GHQ, General Health Questionnaire; IES, Impact of Event Scale; T1, assessment at around 16 weeks gestational age.

For maternal distress assessed at T2, bivariate analyses indicated no significant associations with Q_UVAC_ (see Table 3). Anxiety and arousal reached borderline significance. They were examined in multiple regression models together with covariates. None of the distress measures at T2 predicted Q_UVAC_.

In exploratory regression analyses, looking into a possible pathway for the association between distress levels at T1 and normalized UV volume blood flow, we examined the influence of fetal heart rate (FHR), UA PI, Bw/Pw ratio, and gender-specific gestational-age adjusted placental and birthweight SD-scores (see Table S2, Associations between non-psychometric covariates and normalized umbilical vein blood flow). In univariate analyses, only UA PI and Bw/Pw ratio were significantly and negatively associated with Q_UVAC_. In multiple regression analyses, Bw/Pw ratio, and to a lesser extent placental weight SD score, reduced both size and strength of the association between distress and Q_UVAC_, but did not itself reach statistical significance levels (see Table 4). No effect was seen when using birthweight SD-scores. Adjusting for either FHR or UA PI only resulted in minimal changes in the relation between distress measures and Q_UVAC_ (results not shown).

### Umbilical artery

UA PI at 30 weeks was obtained in 73 pregnancies (mean 1.03, SD 0.16). There were no cases of absent or reversed end-diastolic flow in the UA. In bivariate analyses, the only associations with psychometric scores were at borderline significance level (i.e. T1 EPDS (ordinal), *r*_s_ = −0.23, *P* = 0.051; and T1 depression > mean, *P* = 0.061). P-values for intrusion at either time point varied between 0.41 and 0.58. When examined in multiple linear regression models as predictors for UA PI, after adjustment for FHR, neither EPDS nor depression scores reached *P*<0.10 (results not shown).

### Uterine arteries

UtA PI at 30 weeks was obtained in 74 women (median 0.69, interquartile range 0.61–0.83). We found no significant associations with any of the distress measures, neither in bivariate nor in multiple linear regression analyses, with p-values ranging from 0.13 to 0.95 (results not shown).

## Discussion

We aimed to investigate the effect of several types of maternal psychological distress at different time points in pregnancy on the placental circulation in third trimester.

As expected, we did not find any significant associations between UA or UtA PI and maternal symptoms of anxiety, depression, or distress related to the health of the fetus. Some studies have reported increased vascular resistance measures in the UA [6] or UtA in maternal distress or anxiety [7], [8]. The majority of studies, including a large study by Harville *et al.*, have not found associations between several key types of maternal distress and resistance measures in either the UA [7], [8], [10]–[13] or the UtA [9]–[13]. While these studies focused on low-risk, unselected, or psychiatric populations, we focused on women with a specific, pregnancy-associated and potentially traumatic event prior to the current pregnancy. Despite the high levels of maternal distress in second and third trimester, we could not demonstrate any increase in vascular resistance measures on either the maternal or fetal side of the placental circulation, supporting the negative conclusions from the majority of previous studies.

For normalized UV blood flow volume measured in third trimester, we found an association with several types of maternal psychological distress in second trimester, i.e. symptoms of depression, intrusive distress concerning the health of the fetus, and psycho-physiological arousal. Higher distress levels were associated with the fetus receiving more rather than less blood than expected for its size. Our current results therefore do not support the popular hypothesis that reduced placental blood flow is a pathway by which maternal distress may affect fetal growth and development negatively. However, our study group was selected for a specific preconceptional life event, and findings may not apply to pregnant women without such a history.

Finding increased normalized UV volume blood flow was surprising, since this has been associated with augmented fetal growth [36]. In our study, birthweight SD-score and Q_UVAC_ were not correlated. However, volume flow does not necessarily equal nutrient supply or tissue growth. Hypothetically, several mechanisms could increase UV volume blood flow without simultaneously affecting growth: increased fetal cardiac output, for example through higher FHR or blood pressure; altered distribution of the cardiac output between the fetal body and the placenta; lower vascular resistance in the umbilical cord or the placenta; increased absolute placental volume; and reduced “placental efficiency” (i.e. lower Bw/Pw ratio) [37]. Our study was not originally designed to investigate these mechanisms, and only limited exploratory analyses were possible. Neither adjusting for FHR as a component of cardiac output, nor for UA PI as an indicator of placental vascular resistance, changed the association between distress measures and UV volume flow. Bw/Pw ratio was inversely correlated with normalized UV volume flow. When adjusted for, this variable reduced effect size and significance level of the association between distress and Q_UVAC_. A lower Bw/Pw ratio could be due to a relatively increased placental weight, reduced birthweight, or a combination of both. While adjusting for placental weight SD-score showed a similar effect as Bw/Pw ratio, no effect was seen when using birthweight SD-score. This could indicate that increased placental weight rather than reduced fetal growth plays a role in the association between antenatal psychological distress and increased fetoplacental volume flow.

Due to the small sample size and the post-hoc nature of these analyses, we consider these results to be exploratory and hypothesis generating. However, it has previously been suggested that the placenta may adapt its size, surface area, morphology, or function as a response to environmental stressors and unfavorable conditions [18], [38]. A higher placental weight – both absolute and relative to birthweight – has been observed after dietary restrictions and exposure to synthetic stress hormones in early pregnancy [19], [39], [40]. A recent population-based study found a higher placental weight with increasing antenatal life stress scores, with 57% of the association between stress and placental weight being independent of a related variation in birthweight [41].

The association with UV volume flow was found for distress scores in second but not third trimester. Due to the rapid development of the placenta and the fetal cardiovascular system in the first months of pregnancy, this period may be especially susceptible to the effects of maternal stress on these processes [18], [19], [38]. Perinatal loss or termination of pregnancy due to fetal malformation is associated with persistently increased levels of psychological distress, often extending into subsequent pregnancies [15], [42], [43]. Similar findings apply to parents of children with congenital malformations [16]. Although we did not obtain measurements before pregnancy, it seems reasonable to assume that distress levels in our study group were elevated periconceptionally; the high prevalence of significant clinical distress at T1 supports this assumption.

In contrast with our present findings, in our previous study in women without malformation in a previous offspring we reported a possible reduction of UV blood flow in those with higher IES intrusion scores at 30 weeks [13].This discrepancy could be due to “false positive” findings in our previous paper, for example through chance, multiple analyses, or measurement errors. Another possibility is a “false negative” finding in our current study, due to small sample size. The study group was less homogeneous with respect to some potential confounders (e.g. smoking), while the small sample size and the infrequent occurrence of these variables precluded statistical adjustment. However, the previous study had a lower prevalence of distress, in particular depressive symptoms. The wider distribution and higher levels of distress scores in the current study may compensate for some of the sample size limitations.

Other explanations are possible. The most important differences between the two studies were the type and the timing of exposure. As discussed above, timing of significant distress in the vulnerable early phase of pregnancy might have an opposite effect on fetoplacental volume blood flow, possibly as the result of an adaptive mechanism. In addition, the participants had undergone a potentially traumatic life event, and had increased levels of psycho-physiological arousal. Not only could the new pregnancy act as a constant reminder, resulting in chronic stress, but trauma-related cues may provoke exaggerated physiological responses [44]. In the previous study group, problems with the health of the fetus were hypothetical; high intrusion levels might rather have been a marker of a tendency to anxiousness and worry, leading to different physiological responses.

### Strengths and limitations

The strength of this study lies in the type of the exposure, which allowed us to prospectively study the effects of a naturalistic, preconceptionally timed and pregnancy-specific significant stressor. Due to the timing and characteristics of the exposure, the stress response and physiological adaptation may differ from pregnancies not exposed to this particular stressor. However, for this reason the findings may not be applicable to other populations, making it more difficult to confirm or reject the findings from our previous study. Thus, while our study contributes to the understanding of the effects of psychological distress in pregnancy, it also raises new questions and hypotheses. Another strength is the use of several widely used and validated psychometric instruments, assessing different key types of psychological distress. However, due to the inter-correlation of distress measures in this study, it is difficult to disentangle the effect of anxiety and depression per se. The small sample size constitutes a significant limitation in this observational study. It necessitated use of the mean rather than established cut-off levels in order to retain statistical power. Multiple testing, exaggerated effect size, and limited examination of confounding, modifying, and mediating effects are other challenges in small samples. As also discussed in our previous study, corroboration of these results is therefore required.

In conclusion, in women with a previous fetus or child with a congenital malformation, we found that higher distress levels in the first half of pregnancy were associated with a third trimester fetoplacental blood flow that was higher than expected for the size of the fetus. These results do not support the hypothesis that maternal distress affects birthweight through reduced placental circulation. Our study was not powered to examine the effect of antenatal maternal distress on fetal and placental growth and development. However, exploratory post-hoc analyses suggested that the role of placental weight in early antenatal distress could be an area of interest worth investigating further.

## Supporting Information

Figure S1 **Association between IES intrusion score and normalized umbilical vein volume blood flow (Q_UVAC_; ml/min/cm) (n = 65).** Scatter plot for IES intrusion subscale score at 16 weeks and umbilical vein volume blood flow, normalized for fetal abdominal circumference (Q_UVAC_; ml/min/cm) at 30 weeks.
(DOC)

Table S1 **Psychological distress scores above clinical cut-off levels at 16 and 30 weeks of gestational age.**
(DOC)

Table S2 **Associations between non-psychometric covariates and normalized umbilical vein volume blood flow (Q_UVAC_; ml/min/cm) (n = 65).**
(DOC)
