# Supplementary material for: Maternal Psychological Distress and Placental Circulation in Pregnancies after a Previous Offspring with Congenital Malformation
Source: PLoS One. 2014 Jan 27;9(1):e86597. doi: 10.1371/journal.pone.0086597 (PMC3903559; doi:10.1371/journal.pone.0086597)
Supplement: Figure S1 — Association between IES intrusion score and normalized umbilical vein volume blood flow (QUVAC; ml/min/cm) (n = 65). Scatter plot for IES intrusion subscale score at 16 weeks and umbilical vein volume blood flow, normalized for fetal abdominal circumference (QUVAC; ml/min/cm) at 30 weeks. (DOC) [file pone.0086597.s001.doc]

**Figure S1.** Association between IES intrusion score and normalized umbilical vein volume blood flow (Q_UVAC_; ml/min/cm) (n = 65).

Scatter plot for IES intrusion subscale score at 16 weeks and umbilical vein volume blood flow, normalized for fetal abdominal circumference (Q_UVAC_; ml/min/cm) at 30 weeks.

IES, Impact of Event Scale; Q_UVAC_, umbilical vein volume blood flow, normalized for fetal abdominal circumference (ml/min/cm); r(s), Spearman’s rank correlation coefficient.
