# Supplementary material for: Maternal Psychological Distress and Placental Circulation in Pregnancies after a Previous Offspring with Congenital Malformation
Source: PLoS One. 2014 Jan 27;9(1):e86597. doi: 10.1371/journal.pone.0086597 (PMC3903559; doi:10.1371/journal.pone.0086597)
Supplement: Table S1 — Psychological distress scores above clinical cut-off levels at 16 and 30 weeks of gestational age. (DOC) [file pone.0086597.s002.doc]

**Table S1.** Psychological distress scores above clinical cut-off levels at 16 and 30 weeks of gestational age.

|  | **T1 (16 weeks), n = 73**^a^ | **T2 (30 weeks), n = 74** |
| --- | --- | --- |
| **General Health Questionnaire** |  |  |
| Sum Likert score > mean | 33 (45) | 32 (43) |
| Sum case score ≥6 | 44 (60) | 30 (40) |
| **Edinburgh Postnatal Depression Scale** |  |  |
| Sum score ≥10 | 15 (20) | 10 (14) |
| **Impact of Event Scale** |  |  |
| Intrusion ≥9 | 61 (84) | 40 (56) |
| Intrusion ≥19 | 24 (33) | 15 (20) |
| Avoidance ≥9 | 23 (31)^b^ | 15 (21) |
| Avoidance ≥19 | 6 (8)^b^ | 2 (3) |
| Arousal ≥9 | 35 (49)^b^ | 16 (22) |
| Arousal ≥19 | 4 (6) ^b^ | 6 (8) |

Data are presented as *n* (%).

^a^Missing n = 1 (1 questionnaire set missing).

^b^n = 72 (1 questionnaire page left blank).
