# Supplementary material for: Maternal Psychological Distress and Placental Circulation in Pregnancies after a Previous Offspring with Congenital Malformation
Source: PLoS One. 2014 Jan 27;9(1):e86597. doi: 10.1371/journal.pone.0086597 (PMC3903559; doi:10.1371/journal.pone.0086597)
Supplement: Table S2 — Associations between non-psychometric covariates and normalized umbilical vein volume blood flow (QUVAC; ml/min/cm) (n = 65). (DOC) [file pone.0086597.s003.doc]

**Table S2.** Associations between non-psychometric covariates and normalized umbilical vein volume blood flow (Q_UVAC_; ml/min/cm) (n = 65).

|  | **Q_UVAC_** | | |
| --- | --- | --- | --- |
|  | **unadj. B** | **95% CI** | **P** |
| Maternal age (years) | –0.012 | –0.106, 0.083 | 0.81 |
| Parity (≥1) | 0.531 | –0.441, 1.502 | 0.28 |
| Smoking (yes) | 0.007 | –1.581, 1.595 | 0.99 |
| Assisted fertilization (yes) | –1.098 | –2.838, 0.641 | 0.21 |
| BMI at T2 | 0.064 | –0.043, 0.172 | 0.24 |
| Gestational age at T2 (weeks) | 0.413 | –0.218, 1.043 | 0.20 |
| Ultrasonographer | –0.070 | –1.435, 1.294 | 0.92 |
| Fetal gender (girl) | –0.033 | –0.874, 0.808 | 0.94 |
| Fetal heart rate (beats/minute) | 0.014 | –0.038, 0.065 | 0.60 |
| **Post-hoc analyses** |  |  |  |
| Umbilical artery PI | –2.725 | –5.268,–0.183 | 0.036 |
| Birthweight SD score^a^ | –0.012 | –0.518, 0.494 | 0.96 |
| Placental weight SD score^a^ | 0.275 | –0.116, 0.665 | 0.16 |
| Birthweight/placental weight ratio | –0.523 | –1.000, –0.046 | 0.032 |

^a^Gender- and gestational-age adjusted.

B, regression coefficient; BMI, body mass index (kg/m^2^); PI, pulsatility index; Q_UVAC_, normalized umbilical vein volume blood flow (ml/min/cm); T2, assessment at 30 weeks gestational age; UA, umbilical artery.
